# Association among self-compassion, childhood invalidation, and borderline personality disorder symptomatology in a Singaporean sample

**DOI:** 10.1186/s40479-017-0075-3

**Published:** 2017-11-28

**Authors:** Shian-Ling Keng, Yun Yi Wong

**Affiliations:** 1 Division of Social Sciences, Yale-NUS College, Singapore, Singapore; 2 Psychological Studies Academic Group, National Institute of Education, Nanyang Technological University, Singapore, Singapore; 3 Elm College Office, 12 College Ave West, #01-201, Singapore, 138610 Singapore

**Keywords:** Self-compassion, Borderline personality disorder, Childhood invalidation

## Abstract

**Background:**

Linehan’s biosocial theory posits that parental invalidation during childhood plays a role in the development of borderline personality disorder symptoms later in life. However, little research has examined components of the biosocial model in an Asian context, and variables that may influence the relationship between childhood invalidation and borderline symptoms. Self-compassion is increasingly regarded as an adaptive way to regulate one’s emotions and to relate to oneself, and may serve to moderate the association between invalidation and borderline symptoms. The present study investigated the association among childhood invalidation, self-compassion, and borderline personality disorder symptoms in a sample of Singaporean undergraduate students.

**Methods:**

Two hundred and ninety undergraduate students from a large Singaporean university were recruited and completed measures assessing childhood invalidation, self-compassion, and borderline personality disorder symptoms.

**Results:**

Analyses using multiple regression indicated that both childhood invalidation and self-compassion significantly predicted borderline personality disorder symptomatology. Results from moderation analyses indicated that relationship between childhood invalidation and borderline personality disorder symptomatology did not vary as a function of self-compassion.

**Conclusion:**

This study provides evidence in support of aspects of the biosocial model in an Asian context, and demonstrates a strong association between self-compassion and borderline personality disorder symptoms, independent of one’s history of parental invalidation during childhood.

## Background

Borderline personality disorder (BPD) is a severe condition characterized by dysregulated affect, cognition, behaviors, and interpersonal relationships [1]. Several symptoms of BPD include fear of abandonment, unstable and intense relationships characterized by fluctuations between idealization and devaluation of others, difficulty controlling anger, and chronic feelings of emptiness. BPD is known as one of the most challenging disorders to treat, in part due to the high prevalence of nonsuicidal self-injury and suicide attempts in this population [2, 3]. While there are several developmental models of BPD [4, 5], one of the most dominant models is Linehan’s biosocial theory of BPD [6]. According to the biosocial model, BPD is characterized by marked emotion dysregulation, which arises from a transactional relationship between pre-existing emotional vulnerability (characterized by emotional sensitivity, reactivity, and slow return to baseline) and an invalidating childhood environment. Invalidation may occur in a variety of forms; broadly speaking however, an invalidating environment is one in which a child’s inner experience and expression of emotions, thoughts, and behaviors are frequently criticized, trivialized, ignored, and/or punished. In support of the biosocial theory, various studies have demonstrated an association between childhood invalidation and development of BPD symptoms. Compared to both clinical and non-clinical controls, there is a higher incidence of reported childhood psychological abuse, physical abuse, and neglect among BPD patients [7–9]. Besides severe forms of invalidation such as childhood maltreatment, studies also suggest that parental contradictory communication patterns [10], absence of maternal protection [11], and parental overprotection without affection [12] as perceived by individuals with BPD were associated with the development of BPD pathology. Most of the existing studies were conducted in North American or European settings, which limit the generalizability of their findings to other cultural contexts, such as Asia.

### BPD in Asia

Few studies to date have examined components of the biosocial theory in Asia. Huang et al. [13] recruited a sample of 400 Chinese participants, and found that compared to individuals with other personality disorders and those without personality disorders, individuals who received a BPD diagnosis reported higher levels of parental physical, emotional, and sexual abuse. In another study, Zhang et al. [14] examined a sample of 1402 Chinese patients from an outpatient counselling center, and found that BPD symptomatology was positive associated with childhood emotional, physical and sexual abuse, as well as childhood emotional and physical neglect. While these findings provide some support for the biosocial model, none of the studies specifically assessed the construct of *childhood invalidation* in relation to BPD symptomatology. Further, the fact that Asian cultures tend to emphasize interdependence, emotion control, and hierarchy [15, 16] might imply a high level of invalidation experienced at the individual and/or collective level. While the present study was not set up to provide direct comparison between cross-cultural samples, we aimed to provide a preliminary investigation of the association between childhood invalidation and BPD symptoms in the Singaporean context – a multicultural Asian society influenced by Confucius values as well as other Southeast Asian heritages such as Malay and Indian cultures [17].

### Self-compassion and BPD

Beyond the issue of cross-cultural applicability of the biosocial model, it is important to examine factors that may moderate the association between childhood invalidation and BPD symptomatology. Several factors that have received research attention include affective dysfunction and social support. In one study, affective dysfunction was found to moderate the association between emotional abuse and childhood BPD symptoms, with emotional abuse predicting BPD features only among children with high (versus low) affective dysfunction [18]. Consistent with this study, research has demonstrated that Tryptophan Hydroxylase I (TPH-1) gene – a gene implicated in the serotonergic stress response pathway- moderated the association between childhood abuse and diagnosis of BPD [19]. Another study examined whether social support would moderate the association between childhood sexual abuse and borderline personality features, but did not find support for the moderation effect [20]. Beyond these studies, little work has investigated whether adaptive personality traits may influence the association between invalidation and BPD symptoms. In this study, we were interested in examining self-compassion as a potential correlate of BPD symptoms, as well as moderator of the association between childhood invalidation and BPD symptoms.

A construct originating from Buddhist teachings, self-compassion refers to the tendency to be moved by one’s suffering, such that one yearns to reduce one’s suffering and to treat oneself with kindness and empathy [21, 22]. Neff [21, 22] conceptualizes self-compassion as consisting of three aspects: 1) self-kindness, referring to the ability to relate to oneself kindly; 2) common humanity, which refers to the acknowledgement that setbacks and imperfection are inevitable among all human beings, as opposed to feeling isolated during times of failure; and 3) mindfulness, which refers to being accepting and aware of both negative and positive experiences, as opposed to over-identifying with one’s inner experiences. Depending on context, self-compassion can be conceptualized as a personality trait, referring to the general tendency of an individual to adopt an attitude of compassion towards him- or herself in everyday life [21, 22], a momentary state of being kind to oneself [23], or a strategy of coping with difficult experiences (e.g., intentionally extending wishes of loving-kindness towards oneself when encountering an experience of failure [24]).

As a personality trait, self-compassion has been associated with reduced symptoms of depression and anxiety across multiple contexts, ranging from academic to interpersonal domains [21, 23, 25]. Self-compassion has also been found to correlate with greater well-being in both adolescents and older adults [26, 27]. Among clinical populations, depressed patients have been found to demonstrate lower levels of self-compassion compared to non-depressed individuals, even after controlling for depressive symptoms [28]. Further, the association between self-compassion and depressive symptoms was mediated by shame, symptom-focused rumination, and cognitive and behavioral avoidance [28, 29]. In one study, a brief self-compassion manipulation resulted in decreases in shame and negative affect compared to a control condition [29]. These findings are consistent with the idea that self-compassion serves as a direct antidote to shame [30], which has been proposed to be a core emotion underlying BPD [31]. A meta-analysis found a large effect size (*r* = .54) for the relationship between self-compassion and psychopathological symptoms, particularly depression, anxiety, and stress [32]. Taken together, the findings suggest a strong relationship between self-compassion and psychological health, and point to the role of self-compassion in reducing maladaptive, transdiagnostic emotional and cognitive processes, such as shame, rumination, and avoidance. Little work however has directly examined the association between self-compassion and symptoms of BPD. Given that BPD is characterized by similar maladaptive cognitive processes that have been found to be impacted by self-compassion [31, 33], we predicted that self-compassion would be negatively correlated with BPD symptoms.

Further, there is evidence indicating that self-compassion may moderate, or attenuate emotional reactions to adverse events. For example, Leary et al. [23] found that individuals with high self-compassion showed less negative behavioral and emotional reactions when imagining distressing events, compared with less self-compassionate individuals. Further, people with greater self-compassion demonstrate the ability to acknowledge their role in negative situations without feeling overwhelmed by negative emotions [23]. In another study, self-compassion, relative to self-esteem, predicted greater decreases in anxiety after participants were exposed to an ego-threat (i.e., thinking about their greatest weakness) in a laboratory setting [34]. Among patients with major depressive disorder, a brief self-compassion manipulation was found to be more effective than reappraisal in downregulating depressed mood, particularly at high levels of baseline depressed mood [24]. Overall, these findings highlight the potential role of self-compassion in moderating individuals’ reactions to experiences of invalidation. In the context of BPD, adopting a self-compassionate perspective may help lower one’s tendency to internalize feelings of shame, or self-invalidation that that may result from repeated experiences of invalidation [6].

Further, there is evidence that self-compassion is associated with use of more adaptive emotion regulation styles. For example, self-compassion was found to predict greater emotional processing, as well as lower rumination, thought suppression, and catastrophizing across both cross-sectional and laboratory studies [22, 23]. The common humanity facet of self-compassion may also support the ability to reframe distressing life circumstances as part of what all humans experience [21]. Considering the role of self-compassion in moderating reactions to aversive events and promoting adaptive emotion regulation, it is plausible that high levels of self-compassion may predict a weaker association between experiences of invalidation and development of BPD symptoms. To date, no study has yet examined whether trait self-compassion may moderate the relationship between childhood invalidation and BPD symptoms.

### The present study

The present study aimed to examine the association among self-compassion, childhood invalidating environment, and BPD symptoms in a sample of Singaporean undergraduate students. Based on previous research, it was hypothesized that an invalidating childhood environment would be positively correlated with BPD symptomatology. It was also hypothesized that self-compassion would be inversely correlated with BPD symptomatology. We further predicted that self-compassion would moderate the relationship between an invalidating childhood environment and BPD symptomatology. Specifically, the relationship between an invalidating childhood environment and BPD symptomatology was expected to be weaker among those with higher levels of self-compassion, and vice versa among those with lower levels of self-compassion. In this study, we adopted a dimensional perspective of BPD symptoms and recruited on a nonclinical sample of college students, as young adulthood represents a developmental period whereby the symptoms of BPD tend to begin to emerge [35].

## Methods

### Participants

The sample (*N* = 290; 72% female) consisted of undergraduate students recruited from the research participant pool at the National University of Singapore (NUS). Following Reeves et al. [35], the study adopted a dimensional perspective of BPD symptoms and recruited on a nonclinical sample of college students, as young adulthood represents a developmental period whereby the symptoms of BPD tend to begin to emerge. There were no inclusion and exclusion criteria. The age of the participants ranged from 18 to 31 years (*M* = 19.93, *SD* = 1.51). With regards to ethnicity, 89.7% identified as Chinese, 5.2% identified as Indian, 3.1% identified as Malay, 0.7% identified as Eurasian, and 1.4% identified as “Others”.

### Procedure

Participants were recruited to this study through an advertisement entitled “Emotional Experiences in Daily Life: A Survey Study”. Participants expressing interest in the study were invited to a research laboratory and completed a battery of self-report questionnaires, which were all administered in English (see measures section).^1^ The laboratory session lasted approximately 30 min. Participants were given course credit points for their participation. The study was approved by NUS’ Institutional Review Board.

### Measures

#### Demographic data

A demographic form was administered to collect information regarding participants’ age, gender, and ethnicity.

#### Invalidating childhood environment

The Invalidating Childhood Experiences Scale (ICES) [36] was used to assess perceived parental invalidation prior to the age of 18. The ICES is a two-part self-report measure. For the first part of the ICES, participants were asked to retrospectively provide both paternal and maternal ratings on 14 items using a 5-point Likert scale. A composite score was calculated for each parent, with scores ranging from 14 to 70. The means of the total scores from the paternal and maternal subscales were then calculated so as to obtain a combined parental invalidation score. A higher score would indicate higher perceived parental invalidation. The second part of the ICES comprises four descriptions depicting the three invalidating family environment types, namely typical, perfect and chaotic, as well as one validating family environment type, as outlined by Linehan [6]. For the purpose of this study, only the first part of the ICES was used. The ICES has demonstrated excellent psychometric properties, with good internal consistencies of .80 for paternal invalidation and .77 for maternal invalidation in a clinical sample [36], and likewise good internal consistencies of .88 for paternal invalidation and .90 for maternal invalidation in a non-clinical sample [37]. For this study, the scale’s Cronbach’s alpha was .80 for paternal invalidation and .81 for maternal invalidation.

#### Self-compassion

The Self-Compassion Scale (SCS) [21] was administered to assess participants’ tendency to be self-compassionate during times of stress or setbacks. The SCS measures the positive and negative aspects of the three dimensions of self-compassion: 1) self-kindness versus self-judgment, 2) common humanity versus isolation, and 3) mindfulness versus over-identification. Participants were asked to rate 26 items on a 5-point Likert scale. Item scores were reversed as appropriate, and then averaged to create an overall self-compassion score. The scale has demonstrated good construct validity, internal consistency (α = .92), and test-retest reliability (*r* = .93) [21]. In the present study, the scale’s Cronbach’s alpha was .89.

#### BPD symptomatology

The Personality Assessment Inventory-Borderline Features Scale (PAI-BOR) [38] was administered to assess BPD symptoms. The PAI-BOR is commonly used as a screening tool that measures four components of BPD symptomatology, namely 1) affective instability, 2) identity problems, 3) negative relationships, and 4) self-harm. Participants were asked to give ratings on a 4-point Likert scale for each of the scale’s 24 items. For the current study, only the total scores, ranging from 0 to 72, were calculated for use in the analyses. Higher scores, which denote greater severity of BPD symptomatology, have been shown to distinguish BPD individuals from those with other diagnoses such as mood disorders, anxiety, substance abuse disorders, and antisocial personality disorder [39]. The PAI-BOR has also demonstrated good test-retest reliability (*r* = .86) [38], as well as good convergent and discriminant validity in both non-clinical and clinical samples [38, 40]. The Cronbach’s alpha of the scale in this study was .86.

## Results

Prior to data analyses, data screening was done to check for any violations of normality. The data was considered normal as they fall within the recommended limits for regression analyses (i.e. within |3| for skewness and within |10| for kurtosis) [41]. The data was also checked to ensure that there was no multicollinearity between the predictor variables. Nine outliers were detected using boxplots, and these values were excluded from subsequent analyses. Hence, the final sample size used for the regression analyses was 281.

The means, standard deviations, and Pearson’s *r* correlations were calculated for all the variables (see Table 1). As hypothesized, there was a significant positive correlation between an invalidating childhood environment and BPD symptomatology, *r =* .27, *p* < .01. Invalidating childhood environment was significantly and negatively correlated with trait self-compassion, *r =* −.24, *p* < .01. As predicted, there was also a significant negative correlation between self-compassion and BPD symptomatology, *r =* −.60, *p* < .01, with a large effect size.

**Table 1:** Means, Standard Deviations, and Intercorrelations for All Study Variables

Variable	1. ICES	2. SCS	3. PAI-BOR
1. ICES	–		
2. SCS	−.24^**^	–	
3. PAI-BOR	.27^**^	−.60^**^	–
*M*	29.64	3.00	27.02
*SD*	6.83	.52	9.29

*Note. ICES* Invalidating Childhood Environment Scale, *SCS* Self-Compassion Scale, *PAI-BOR* Personality Assessment Inventory-Borderline Subscale

^**^ *p* < .01

A series of hierarchical regressions were conducted to test the moderating effect of trait self-compassion on the relationship between an invalidating childhood environment and BPD symptomatology. First, the predictor variables (invalidating childhood environment, self-compassion) were mean-centered. Second, using BPD symptomatology as the criterion variable, invalidating childhood environment and self-compassion were entered as predictors in Step 1 of the regression. In Step 2, the interaction term of invalidating childhood environment × self-compassion was entered. Table 2 shows a summary of the results.

**Table 2:** Summary of Regression Results of the Moderating Effect of Self-Compassion on the Relationship between Invalidating Childhood Environment and BPD Symptomatology

	Δ*R* ^*2*^	*B*	*SE*	β	*t*	*p*
Step 1	.382					
ICES		.182	.066	.134	2.766	.006
SCS		−10.171	.862	−.573	−11.801	< .001
Step 2	.000					
ICES		.183	.066	.134	2.766	.006
SCS		−10.132	.871	−.570	−11.638	< .001
ICES × SCS		.042	.119	.017	.351	.726

*Note*. *ICES* Invalidating Childhood Environment Scale, *SCS* Self-Compassion Scale

In Step 1, invalidating childhood environment and self-compassion accounted for a significant amount of variance in BPD symptomatology, Δ*R* ^*2*^ = .382, *F*(2, 278) = 85.96, *p* < .001. There was a significant positive relation between an invalidating childhood environment and BPD symptomatology, *β* = .134, *p* = .006. There was also a significant negative relation between self-compassion and BPD symptomatology, *β* = −.57, *p* < .001.

Step 2 of the analysis showed that adding the interaction term of of self-compassion and childhood invalidation did not significantly improve the regression model, Δ*R* ^*2*^ = .00, *F*(3, 277) = 57.16, *p* = .73. Thus, contrary to expectation, self-compassion did not significantly moderate the relationship between an invaliding childhood environment and BPD symptomatology.

## Discussion

The present study aimed to examine the association among self-compassion, childhood invalidating experiences, and BPD symptoms in a sample of Singaporean undergraduate students. The study found that childhood invalidation was positively associated with BPD symptoms, whereas trait self-compassion was negatively related to BPD symptoms. Contrary to expectation, the relationship between an invalidating childhood environment and BPD symptomatology did not vary as a function of one’s level of trait self-compassion.

The finding that a higher level of childhood invalidation was related to greater BPD symptomatology is consistent with Linehan’s biosocial theory [6], which posits that the interactions between a pre-existing emotional vulnerability and an invalidating environment result in later development of symptoms of BPD. A higher level of trait self-compassion was found to be strongly related to lower BPD symptomatology. This result complements previous findings that self-compassion is linked with decreased anxiety, depression, self-criticism and feelings of shame, as well as greater psychological health [21, 23, 25, 42]. The results suggest that having a kind and mindful attitude toward one’s unpleasant experiences may be related to greater affective stability and a lower tendency to engage in maladaptive behaviors commonly seen in the context of BPD. Further, a non-judgmental stance toward oneself likely increases self-acceptance and a sense of self-worth, which may facilitate the formation of a coherent sense of self, in contrast to the symptom of disturbed identity often seen among patients with BPD.

The study further found that trait self-compassion did not moderate the relationship between childhood invalidation and BPD symptoms; rather, self-compassion predicted lower BPD symptoms equally across both high and low levels of childhood invalidation. The findings suggest that self-compassion may be more strongly associated with BPD symptoms than previously thought. This finding is consistent with the current literature, which shows that self-compassion is associated with adaptive emotion regulation and coping across both clinical and nonclinical populations [43–46], who may experience varying degrees of invalidation in their developmental experiences. The finding suggests that self-compassion acts as a general correlate of psychological health, likely through promoting healthier coping strategies (e.g., less avoidance [25]), which in turn is associated with lower symptoms of BPD. For individuals who underwent repeated experiences of invalidation, adopting a self-compassionate perspective or predisposition may help lower the degree of shame and self-invalidation that may result from these experiences [6, 29]. Clinically, the findings point to the potential benefits of incorporating self-compassion training into existing interventions for BPD, regardless of the degree of invalidation reported by patients [47, 48].

Existing research showed that self-compassion is an inner resource that can be trained and developed over time [49]. In fact, to some extent, self-compassion is already featured in selected existing interventions for BPD, such as dialectical behavior therapy (DBT [6]). For example, the skill of radical acceptance in DBT involves adopting an attitude of accepting and embracing difficult experiences as they are (opposed to resisting or struggling against them), which may over time result in a kinder way of relating to one’s experiences. Recent developments in DBT also included a greater emphasis on the value and practice of loving-kindness meditation, which involves intentionally generating wishes of loving-kindness to oneself and others [50]. Gilbert and Proctor [51] suggested that self-compassion training could prime an individual to access his or her self-soothing system more easily. The researchers developed the compassionate mind training (CMT) program, in which imagery and letter-writing techniques are used to generate compassionate warmth and understanding toward the self. Attendance of the program was associated with a significant increase in self-soothing ability, along with decreases in self-criticism, shame, depression, anxiety, inferiority, and submissive behavior in a sample of individuals with high shame and self-criticism [51]. Similarly, a pilot study on compassion-focused therapy, which included elements of CMT, demonstrated significant improvements in shame, sense of inferiority, self-reassuring abilities, depression, and stress in a sample of patients with personality disorders and a history of chronic complex trauma [52]. Therefore, explicit training in self-compassion may be beneficial particularly for individuals with a history of childhood maltreatment or invalidation. Future research should examine the effects of self-compassion training on individuals with BPD symptoms. Given that self-compassion training can be delivered in a variety of modalities (e.g., loving-kindness meditation or letter writing), it would also be helpful for future research to examine modes of treatment delivery that would be most effective for these individuals.

There are several strengths to this study. The study recruited a relatively large sample and included several ethnicities in Singapore. The study is also among the first to investigate aspects of BPD’s biosocial model in the Singaporean context, with the findings lending support to the validity of the model in the local cultural context. In particular, the findings indicate that childhood invalidation is a common correlate of BPD symptoms in both Singaporean and other cultural contexts [9–11, 35]. Future research should adopt cross-cultural samples to examine differences with regards to the degree of, as well as association among self-compassion, invalidation, and BPD symptoms. Preliminary research suggests that there are country-level differences in levels of self-compassion among the United States, Taiwan, and Thailand, with self-compassion being lowest in Taiwan [53]. Among these countries, Singapore, with Chinese constituting the majority of its population, is arguably most similar to Taiwan in terms of its culture. It would be interesting for future research to examine collective levels of self-compassion and invalidation, and their implications on the prevalence and expression of BPD and related symptoms in the local context.

There are some limitations in this study. Importantly, as the study’s design is correlational and cross-sectional, causality cannot be inferred. For instance, it is possible that persons with BPD find it difficult to be self-compassionate, due to emotional dysregulation that stemmed from long-term invalidation during childhood. In other words, self-compassion may not play a causal role in the development of BPD symptoms. Further, our measure of childhood invalidation relies on retrospective recall, and is therefore subject to memory bias. Future research should employ a longitudinal design to examine the temporal association among childhood invalidation, self-compassion, and development of BPD symptomatology, and the interplay among these variables over time. Studies using an experimental design would also be helpful to examine the causal relationship between self-compassion and symptoms of BPD. Second, this study utilized a relatively homogeneous undergraduate sample. Thus, the findings may not be generalizable to other populations. Future research should replicate the findings in a more diverse sample, as well as in a clinical (i.e., diagnosed BPD) sample. Lastly, as the data were obtained using self-report measures, the findings may be attributed to shared method variance. Future research should incorporate multiple modes of assessment (e.g., use of interviews and/or observations) to assess the association among self-compassion, invalidation, and BPD symptoms.

## Conclusions

Overall, the findings from the current study provide support for the association between childhood invalidation and BPD symptoms in an Asian context. The study also showed that self-compassion independently predicts BPD symptomatology, over and above the effects of an invalidating childhood environment. Future research should investigate ways in which various components of the biosocial model, such as pre-existing vulnerability to emotion dysregulation, interact with self-compassion in giving rise to BPD symptoms. It would also be helpful for future research to investigate potential developmental antecedents of self-compassion, such as the degree to which validation is expressed within the family. While further longitudinal or experimental research is necessary to assess the causal relationships among self-compassion, BPD, and invaliding childhood environment, the results of this study demonstrate the role of both self-compassion and childhood invalidation as important correlates of BPD symptoms.

## Footnotes

1 English is widely spoken and one of the official languages in Singapore.
